# Editorial

**Published:** 1853-03

**Authors:** 


### THE MEDICAL EXAMINER.

PHILADELPHIA, MARCH, 1853.

### THE SOUTHERN JOURNAL OF THE MEDICAL AND PHYSICAL SCIENCES.

This is the title of a new bi-monthly, published at Nashville, Tennessee, under the editorial charge of "Drs. Jno. W. King and Wm. P. Jones, in the department of Practical Medicine and Surgery; Dr. R. O. Currey, Chemistry and Pharmacy; and Dr. B. Wood. Dental Surgery." It promises to be a spirited and able addition to the rapidly growing ranks of the American Medical Press.

### THE ESCULAPIAN: A MONTHLY MEDICAL PAPER FOR THE PEOPLE.

We very cordially commend this publication to general favor. Its objects are implied in the title, which we quote above; and they have been carried out, in the two numbers which have come under our notice, with marked discretion and ability. It is edited by D. C. Griswold, M. D., and published for the low subscription of one dollar a year.

### LECTURES ON EXPERIMENTAL PHYSIOLOGY.

Dr. E. Brown-Séquard proposes to give a course of lectures on Experimental Physiology in this city, early in March. It will embrace the Physiology of the Nervous System, with applications to Pathology. He will also give a course to students at the same time.

Both courses will be illustrated by vivisections.

Dr. Brown-Séquard's thorough acquaintance with this department of science, will ensure ample repayment to all who may attend upon his instructions and demonstrations.

